# Oromotor Nonverbal Performance and Speech Motor Control: Theory and Review of Empirical Evidence

**DOI:** 10.3390/brainsci13050768

**Published:** 2023-05-06

**Authors:** Gary Weismer

**Affiliations:** Department of Communication Sciences & Disorders, University of Wisconsin-Madison, Madison, WI 53706, USA; gary.weismer@wisc.edu

**Keywords:** speech motor control, oromotor, nonverbal motor control, dysarthria, speech production, task specificity

## Abstract

This position paper offers a perspective on the long-standing debate concerning the role of oromotor, nonverbal gestures in understanding typical and disordered speech motor control secondary to neurological disease. Oromotor nonverbal tasks are employed routinely in clinical and research settings, but a coherent rationale for their use is needed. The use of oromotor nonverbal performance to diagnose disease or dysarthria type, versus specific aspects of speech production deficits that contribute to loss of speech intelligibility, is argued to be an important part of the debate. Framing these issues are two models of speech motor control, the Integrative Model (IM) and Task-Dependent Model (TDM), which yield contrasting predictions of the relationship between oromotor nonverbal performance and speech motor control. Theoretical and empirical literature on task specificity in limb, hand, and eye motor control is reviewed to demonstrate its relevance to speech motor control. The IM rejects task specificity in speech motor control, whereas the TDM is defined by it. The theoretical claim of the IM proponents that the TDM requires a special, dedicated neural mechanism for speech production is rejected. Based on theoretical and empirical information, the utility of oromotor nonverbal tasks as a window into speech motor control is questionable.

## 1. Introduction

### 1.1. Historical Perspective

The significance of nonspeech, oromotor control for theories of speech motor control, and for the clinical diagnosis and management of speech motor control deficits have long been written about in the literature. Relevant publications extend back in time in the literature at least to the 1940, when Emil Froeschels (1884–1972), one of the original academic speech pathologists, advocated chewing as a therapeutic technique for stuttering and other speech disorders [1,2]. (I use Froeschels as a blurry reference point in time for the beginning of “academic” publications on the topic of oromotor nonspeech postures and gestures and their relationship with speech (and voice) production. An earlier example was published in 1893 by Oskar Guttman in a book titled, *Gymnastics of the Voice, a method for song and speech, also a method for the cure of stuttering and stammering* [3]. This second edition in English, translated from a version (one of at least four), was originally written in German. Guttman, writing on exercises for the tongue, (which he claimed most people regarded as no more than a “helpless lump of meat” (p. 83), suggested back and forth tongue tip movements between the corners of the mouth, slowly at first and gradually ramping up to maximum speed. Examples of other tongue exercises (among 16) included protrusion–retraction cycles and rapid “dotting” of the upper lip with the point of the tongue. Lip exercises included practice for independent movements of the upper and lower lip and a velar exercise of raising and lowering the soft palate with the mouth open wide. Close reading of Guttman’s book suggests it was intended more for singers than speakers, but he clearly meant the oromotor nonverbal exercises to apply equally to speech, as suggested in the subtitle of the book. Other similar manuals and textbooks were written in the late nineteenth and early twentieth centuries. They are written still today). For Froeschels [2], the act of silent (mimed) chewing, especially with exaggerated movements of the tongue and lips, harkened back to the biological origins of modern voice and speech production. Preverbal people discovered that these motions could be superimposed on sound made in the throat, resulting in vocal tract signals that changed with the motions. Chewing, in Froeschel’s view, took a patient back to the most basic of oromotor behaviors, a logical starting point for voice and speech therapy. Froeschels (Ref. [1], p. 127) offered this anecdote as evidence for this view: “…gum chewing played an interesting part in my studies on the influence of chewing on voice and speech. Two years ago, my wife and I made a trip on a Greyhound bus. The drivers changed regularly, and the new driver always addressed the travelers. I could then predict to my wife, without a single error, whether or not the driver chewed gum, making the differential diagnosis from the impression of a hyperfunctional or a hypofunctional voice, on the one hand, or of a normal voice, on the other. The normal voice indicated to me that the man was used to chewing gum”. The shared anatomical structures for chewing and speech production led Froeschels to claim, “In fact, only one single center (in the anterior central convolution of the brain) dominates the movements of chewing and talking. It stands to reason that two different functions cannot be performed at the same time by one single part of the body. From this, we can conclude that, as far as the movements of the mouth are concerned, what has been considered two functions, namely talking and chewing must be one and the same” (Ref. [2], p. 9). (The paper appeared in [2] and is reprinted in Froeschels’ *Selected Papers of Emil Froeschels, 1940–1964* [4]). As discussed below, Froeschel’s views are a variation on the common effector arguments for the relevance of oromotor nonverbal control to speech motor control [5,6]; see the discussion in [7]. Obviously, you *can* chew and speak at the same time, as evidenced by the millions of mothers and fathers who have told their children not to. (A close reading of Froeschels publications on the near identity of motor control for chewing and speech suggests some inconsistencies in his thinking about doing two different things simultaneously with shared effectors. Usually, his view is the one expressed above, but at least one example of a different opinion, that you *can* chew and speak at the same time, can be found in the chewing papers collected by Froeschels [4]. The book also contains papers on deafness, psychoanalysis, stuttering, aphasia, and language characteristics).

Froeschels seemed to regard chewing and speaking, or any other combination of simultaneous oral actions, as subtasks of oral control. This I discuss below in Section 5. Task Specificity.

### 1.2. Purposes of the Present Paper

This essay has several purposes. Because the different purposes are often interconnected, their order as presented in the current section is not meant to correspond exactly with the sequence of sections that follow. Potential relationships between oromotor nonverbal behaviors and matters such as swallowing and sleep apnea are not considered here.

One purpose of this paper is to outline general issues concerning nonspeech oromotor (NSOM) tasks and their relationship with speech production. These considerations include the difference between the differential diagnosis of neurological diseases versus speech production deficits secondary to those diseases. Selected facts concerning speech production in neurologically healthy speakers and speakers with dysarthria are also discussed.

A second purpose is to provide an evaluation of NSOM tasks, also identified throughout this paper as oromotor nonverbal tasks, and how they are thought to relate to speech production. I argue that a significant amount of contemporary research and clinical practice embraces the broad outlines of Froeschel’s view, with potential for more sophisticated and nuanced measurements (see [7], for discussion of the limits of instrumented speech analysis in clinical settings). For example, in many cases, the study and assessment of oromotor nonverbal control does not require an examiner’s perceptual judgment of observed behavior but can be quantified with instrumental techniques if they are available [8]. Componential (presumed single structure) evaluation of oromotor, nonverbal control, either observational or less frequently quantified via instruments, is used in the clinic, as exemplified by tasks such as soundless or syllabic diadochokinesis (DDK) to evaluate both rate and rhythmic control of the lips, tongue, jaw, and vocal fold movements. Quantitative assessment of other behaviors such as maximum force (F) or pressure (P; P = F/unit area) to estimate the strength of individual oral mechanism components and the rate of cyclic movement (such as opening and closing the jaw or wagging the tongue back and forth at the maximum rate or repeating voiced syllables at the maximum rate) are also thought to provide useful information on speech production deficits and their treatment. In short, this section addresses the possibility of reconciling performance on NSOM tasks and speech production phenomena among neurologically healthy speakers and speakers with dysarthria.

As reviewed by Ziegler [9] and Weismer [6], a central assumption of the view that NSOM tasks are relevant to speech motor control is that shared anatomical structures imply some degree of shared control for different functions. The assumption has been made most typically for peripheral structures such as the tongue, lips, and jaw. More recently, it has been pushed upstream in functional imaging research, in which partially overlapping regions of the brain are active for both speech and nonspeech gestures or their sequencing. In either case, the overlap of structure or activity is taken as evidence for a degree of common control (e.g., Refs. [10,11] see critique of this logic in [12], pp. 135–136). In Weismer [6], this is called the “common effectors” fallacy. Herein, I replace “fallacy” with “assumption” to correct the implication of conclusiveness suggested by “fallacy”. The basic question to be addressed is the possibility of reconciling the information derived from NSOM tasks with speech production data.

A third purpose is to provide a sketch of the important distinction between the use of NSOM tasks to diagnose neurological disease versus speech production deficit. To the best of my knowledge, the literature does not contain an in-depth discussion of this issue. I believe that this diagnostic distinction is likely to be at the heart of the potential for NSOM tasks to further understanding of speech production deficits in dysarthria, and more generally to develop and refine theories of speech motor control [13].

A fourth purpose is to identify and evaluate publications that report empirical data on the relationship between oromotor nonverbal tasks and measures of speech production in both neurologically healthy speakers and speakers with motor speech disorders. These tabulated summaries (Table 1 and Table 2) are intended as an extension of Tables II and III in Weismer [6].

Finally, brief consideration is given to cyclic tasks such as silent, rapid sequences of vocal tract opening and closing, or the same task with voiced syllables. Another example is rapid tongue wagging at maximum rate although a speech-like correlate of this NSOM task, unlike the case of silent and voiced syllables, is not obvious. These are examples of diadochokinetic DDK tasks, which have been used extensively in the clinical evaluation of motor speech disorders, as well in the literature on motor speech disorders and in neurotypical speakers and speakers with other kinds of speech disorders; this issue is addressed only briefly because of a recent extensive review of DDK [13].

Is it necessary to write more about this issue? I believe it is, for the following reasons. First, NSOM tasks are used frequently in clinical settings. Since the publications of Ziegler [9,14] and Weismer [6], several publications [15,16,17,18,19,20,21,22,23] point to the frequent use of oromotor nonverbal tasks in clinical settings, while acknowledging the absence of empirical evidence to support their use for speech production. Surveys of the use of NSOM tasks reported by previous researchers [15,17,24], while a decade or more old, are consistent with opinions offered in the studies cited above that the clinical use of NSOM tasks continues with essentially no empirical support. The issue continues to be debated in more recent literature [19,25,26,27].

Much of what follows is congruent with questions raised by Ziegler et al. [7], and the implications of empirical work described in a series of publications. As noted by Ziegler et al., these data raise serious doubts concerning the value of NSOMs in understanding the speech deficit in dysarthria. Theoretical issues also bear on the potential relationships between NSOM tasks and speech production: “…unresolved theoretical issues compel us to challenge the diagnostic value of nonspeech parameters in the clinical assessment of a patient’s motor *speech* impairment. The most general theoretical question is to what extent the organization of motor behavior differs between speaking and other orofacial motor activities…as well as the particularities of motor goal setting and of sensorimotor principles in specifying speech motor functions over other motor functions of the speech musculature” (Ref. [7], p. 21, emphasis added).

## 2. Definitions

### 2.1. Operational Definitions Formulated for the Purposes of This Paper

The following definitions are not meant to improve those for the same or similar terms in other publications.

Several definitions are revisions of ones in Weismer [6]. In his 2017 publication, Maas claimed that a problem with the oromotor nonspeech/speech production controversy is the absence of precise definitions required to frame the debate [26]. Precisely stated (but nevertheless arguable) definitions of the terms, “dysarthria”, “Mayo Clinic approach”, “oromotor nonverbal tasks”, “quasi-speech tasks”, and “explanation” are given in Weismer [6]. Herein, I revise and broaden definitions for “dysarthria”, “oromotor non-speech tasks”, and “quasi-speech tasks”. New definitions are provided for “task specificity”, “apraxia of speech”, and “speech motor control”, which surprisingly (to the author as well) were not formally included in Weismer [6] but see page 329). A brief note follows some of the definitions.

#### 2.1.1. Speech Motor Control

Speech motor control is defined as the goal-directed, nervous system control of speech production by which movements, and their derivatives, effect shapes and configurations of structures throughout the speech mechanism, including laryngeal and respiratory structures, all planned and executed as a function of time to produce sequences of acoustic phonetic events for the purpose of communication. Acoustic phonetic output of the vocal tract is an integral component of speech motor control processes, not separate or subtractable from them. The speech acoustic signal is intertwined with the movements and their derivatives stated above by calibrating the latter with the former, and the former with the latter. *Note*: The claim that the speech acoustic output is the goal of speech motor control is based on the work of Guenther [28], Perkell [29] and Hickok and Poeppel [30]. See Bose and van Lieshout [31] for a different view.

#### 2.1.2. Motor Speech Disorder

A motor speech disorder is a neurologically based disorder of speech motor control in which movements of the upper airway structures (including the velopharynx) and the constrictions and configurations they produce, as well as movements of the laryngeal and respiratory structures, and/or the planning and programming of those movements, are affected in ways that result in a speech acoustic signal that is phonetically and/or prosodically degraded and therefore cause deficits in speech intelligibility and/or naturalness. *Note*: “Neurologically based disorders” are those demonstrated on imaging studies (e.g., as in stroke, or multiple sclerosis) or those inferred from signs and symptoms (e.g., as in Parkinson’s disease or childhood apraxia of speech). Examples of signs of a speech motor control disorder include, but are not limited to, disorders of speaking rate, speech rhythm, sound-segment production, speaking fundamental frequency and/or sentence-level pitch modulation, prominence of syllables for lexical stress, and regulation of speech loudness.

#### 2.1.3. Dysarthria

Dysarthria is a type of motor speech disorder, primarily regarded as an execution deficit resulting from muscle weakness, abnormalities of muscle tone, timing and coordination, in which the speech deficit is secondary to documented neurological damage resulting from a number of different diseases. Different types of dysarthria, developed in the perceptual studies of Darley, Aronson, and Brown [32] and refined by Duffy [33], are thought to be associated with damage to different parts of the nervous system. *Note*: As documented below, a reliable match between speech symptoms and dysarthria type, or between speech symptoms and disease diagnosis, has not received much support in the literature. This calls into question the suggestion of different speech therapies according to dysarthria type [33] as well as the frequent use of dysarthria or disease type as an experimental blocking variable.

#### 2.1.4. Apraxia of Speech

Apraxia of speech (AOS) is a speech planning or programming dysfunction, as in the preparation of a sequence of syllables ahead of their production. AOS is a motor speech disorder in which the speech deficit is not thought to be the result of muscle weakness, tone abnormalities, and so forth. AOS is therefore different from dysarthria, although a simultaneous diagnosis of apraxia of speech and dysarthria can be made in the same patient. In this paper, AOS is not considered in depth.

#### 2.1.5. Oromotor, Nonverbal Tasks (Nonspeech Oromotor (NSOM) Tasks)

Any performance task, absent phonetic goals, in which structures of the speech mechanism—especially those of the upper airway—are measured for any aspect of force production (maximum force, ‘‘fine’’ (submaximal) force, positional or force accuracy and/or hold duration (endurance), stability, repetition rate, impulse production, tone, range of motion, speed of motion, movement repetition rate and regularity, structural shape/configuration (e.g., lip rounding), and/or tracking accuracy). *Note*: The term and its acronym “Nonspeech oromotor” (NSOM) is in frequent use in the literature, most often to describe treatments used in the speech clinic, many of which are manualized for clinician use [34]. Nonspeech tasks performed by speech structures that are not part of the upper airway, such as laryngeal and respiratory structures, have also been studied, but are not discussed here.

#### 2.1.6. Task Specificity

In motor control, task specificity is accomplished by flexible “tuning” of sensorimotor processes within nervous system networks to achieve a specific behavioral action/goal. Task-specific tuning is implemented by differential activity within the motor control network of fiber tracts and/or cortical and subcortical cells connected by these tracts. The tuning is learned and refined with extensive “practice” to achieve the desired goal; in the case of speech production, “practice” is not used in the sense of mastering a musical instrument or sports skill, rather it is embedded in the production of millions of utterances. The goal of speech motor control is to generate an acoustic phonetic output of the vocal tract that serves the purpose of communication. *Note*: This definition does not equate “tunings of networks” with specific structures and/or mechanisms; as discussed in Section 5. Task Specificity.

#### 2.1.7. Quasi-Speech Tasks

Tasks performed, on command, in which structures of the speech mechanism produce phonetic (or phonetic-like sounds, as in sustained vowels or fricatives), single syllables that are not words, diadochokinetic (DDK) tasks using repetition of a single syllable (e.g., /pʌpʌpʌ…/), or syllables with varying phonetic content (e.g., /pʌtʌkʌ…/), or sequences of other novel sounds or phonatory events, which have no lexical or paralinguistic function. Quasi-speech tasks may also include maximum and minimum fundamental frequency (F0), maximum and minimum phonatory intensity as a function of voice F0, F0 glissandos at different rates of change, and a variety of other sounds (e.g., raspberries, hissing sounds, rhythmic sequences of mouth sounds). *Note*. Quasi-speech tasks are difficult to define as the question of what is lexical or paralinguistic is subject to debate. For example, an argument can be made for hissing as lexical and/or paralinguistic, depending on context. The sound of hissing is not a word but may serve a communication purpose just like the word “hiss”. Ziegler et al. [7] refer to these quasi-speech events as non-speech, which in the current opinion is a reasonable way to categorize these productions. *Quasi-speech* is used in this paper for these events instead of non-speech because it is relevant to the idea of a continuum of tasks ranging from clearly not speech, to increasingly more speech-like events until the endpoint, “speech” is reached (see [35] discussion of different DDK tasks as points along such a continuum). The common factor among various quasi-speech tasks is that they require production of an acoustic signal, which may or may not be phonetic. The definition is not meant to apply to prelingual children because they may produce segmental and phonatory behaviors before understanding the directions to perform such tasks. Ziegler et al. [7] refer to repetitive syllable tasks as “nonspeech” tasks. I mostly agree with that classification—DDK tasks are not speech in the sense that they are not composed of sounds meant to be comprehended—but choose to use the narrower term to include other tasks as listed above.

#### 2.1.8. Subsystems

Partitions of peripheral speech mechanism structures conceptualized as independent, functional components of speech production. *Note:* The partitions take various forms in the literature. These are considered further in Section 4. Two Models of Speech Motor Control.

#### 2.1.9. Mechanism

Nervous system processes for control of action; in the case of speech motor control, the neural processes organized and tuned to produce an acoustic signal having phonetic value for communication.

## 3. Preliminary Considerations

### 3.1. Diagnosis of Disease versus Speech Production Deficit

I make a critical distinction in this paper between the potential role of oromotor nonverbal tasks in the diagnosis of disease versus an understanding of global and specific speech production deficits associated with (secondary to) neurological disease (Ref. [36]; see review in [37]). This distinction would be unnecessary if type of dysarthria, or neurological disease, predicted global or specific characteristics of a speech motor control deficit. However, neither dysarthria nor disease type yields such reliable mappings, with possible exceptions in very mild or very severe dysarthria. To date, there is scant evidence that the performance on oromotor nonverbal tasks predicts disease or dysarthria type, or global and specific aspects of a speech motor control disorder. By “global aspects”, I mean (at least) type and severity of dysarthria, based on human judgments (Judgments of dysarthria type, even by SLPs and other trained professionals (e.g., neurologists) are not reliable as demonstrated in several experiments (e.g., Refs. [38,39]). In many experiments in dysarthria, whatever the level of analysis (e.g., kinematic, acoustic perceptual), type of dysarthria is a classification variable, wherein type is determined with the revised Mayo Clinic approach) (e.g., Refs. [38,39,40] or by automated analysis [41]). “Specific aspects” of a speech motor control disorder include the observation of dysfunction at the electromyographic, kinematic, aerodynamic, acoustic, and/or perceptual levels of analysis. Several research and clinical questions arise when considering the potential relationship between oromotor nonverbal performance and specific aspects of speech production. For example, does performance on an oromotor nonverbal task, or a set of such tasks, predict specific aspects of articulatory movement for speech production among speakers with dysarthria and/or acoustic characteristics of segmental production? Or does such performance predict particular substitution/distortion/omission patterns? Most importantly, do oromotor nonverbal tasks have predictive value for speech intelligibility deficits? These predictions can be reversed, as well; for example, does disease or dysarthria type predict performance on oromotor nonverbal tasks?

To the best of my knowledge, experiments to address focused questions such as these have not been conducted or have not been published. Only a handful of studies have explored an association between nonverbal, oromotor performance and speech measurements at any of the levels of analysis noted above. For those that have been reported, the majority failed to find a meaningful association between the two (see Table 1 and Table 2, in Section 6. Empirical Findings). Surprisingly, even fewer publications report comparisons of oromotor, nonverbal performance among different dysarthria or neurological disease types [42,43,44]. These studies are discussed further in the Section 6 “Empirical Findings”. (Here, “diagnose a neurological disease” means the use of the traditional cranial nerve examination to diagnose neurological disease on the basis of oromotor dysfunction, as well as tests of reflexes, limb and hand movements, and imaging studies. However, the distinction between diagnosing a neurological disease versus a dysarthria, or more broadly a disease from the cranial nerve examination is not so straightforward. The type of dysarthria or specific aspects of speech production deficits (as discussed in text) have not been predicted from the results of a cranial nerve examination or performance on any other set of orofacial nonverbal tasks or rapid syllable repetitions [45]. Interestingly, in a 2015 survey of SLPs who were asked to rate which assessments provided the most valuable information on speech intelligibility in adults with dysarthria, the oral mechanism examination, cranial nerve examination, and DDK were each ranked substantially more valuable compared with several standardized tests in which speech is the focus (Ref. [23], see their Figure 4). A similar survey finding was reported for SLTs in the UK [46]).

The issue of diagnosis of disease versus dysarthria type is important in experimental work. For example, dysarthria type is used as a classification variable in many experiments (e.g., [42,47,48], disease type in others (e.g., [49,50,51]). A smaller group of studies use classification variables that are a hybrid of dysarthria and disease types [37]. Dysarthria type, as determined on the basis of the revised Mayo Clinic approach [33], is most often based on judgments made by SLPs and/or neurologists. Dysarthria types are not assigned on the basis of objective rubrics, which in theory could be derived from Darley, Aronson, and Brown [32] and Duffy [33], but rather on global perceptual impressions of speech samples, possibly combined with results from a cranial nerve exam, nonspeech signs (i.e., limb tone), and knowledge of the underlying disease diagnosis. For example, “Two speech-language pathologists (SLPs) concurred the dysarthria type was consistent with the underlying medical diagnosis” (Ref. [37], p. EL210). This is all well and good, but the logic in classifying dysarthria type is somewhat circular because speakers in the dysarthria database used by Lansford et al. [37] were chosen to represent the classic features of the individual dysarthrias [32,33]. Spastic dysarthria, for example, may be inferred from a diagnosis of upper motor neuron disease, ataxic dysarthria from cerebellar disease, and so forth (see example in [37]; related reviews are provided in [36,52]). However, such inferences are not particularly reliable, as indicated previously [38,39,52]. Different approaches to the problem of dysarthria classification have been reported; see [53] (free classification) for an alternate approach to classifying dysarthria type with acoustic measures, and [54] for automatic classification of dysarthria type.

Classification of dysarthria type based on the Mayo Clinic system, or based on a combination of disease type and perceptual analysis, extends to the clinic where it may be used to formulate treatment plans [33] For example, the assumed underlying neuropathologies in flaccid versus spastic dysarthria may suggest an orofacial strengthening program in the former but not the latter [33]. In the case of flaccid dysarthria, the progress of a strengthening program can be tracked quantitatively with measurements derived from instruments such as the Iowa Oral Performance Instrument (IOPI). In spastic dysarthria, on the other hand, strengthening is regarded as contraindicated. Unfortunately, there is no evidence one way or the other for the efficacy of strengthening oral structures to improve outcome measures such as speech intelligibility or speech severity, or “specific” measures of articulatory performance (see Section 3.1, above).

### 3.2. Can Performance on Oromotor Tasks Be Reconciled with Speech Production Deficits in Diseases/Dysarthrias?

This section focuses on the relationship between NSOM tasks and speech production characteristics in dysarthria but is relevant as well to speech motor control in neurotypical speakers. Similar observations on this issue are available in [7].

The challenge of reconciling oromotor nonverbal performance with speech motor control disorders is a recurring finding that across disease/dysarthria groups, many kinematic, acoustic, and perceptual phenomena are similar, not different. Surprisingly, as noted by [36,55], in contrast to studies in which a group of speakers with one type of disease/dysarthria is compared to neurologically healthy speakers, few studies have compared movement and/or acoustic characteristics among groups of people with different types of neurological diseases/dysarthrias.

A study of lip motion for single word utterances produced by speakers with Parkinson’s disease, Huntington’s Disease, cerebellar atrophy, upper motor neuron disease, and control speakers showed, for the most part, similar kinematic measures (amplitude, stiffness, acceleration/deceleration) among the four clinical groups and the control speakers. When significant differences were found, they were typically for comparisons between the group with cerebellar disease and the control speakers [49]. In another study [56], kinematic measures of inter-articulatory coordination between the lips and tongue for /u/ in the word “suit” were essentially the same for control speakers and speakers with amyotrophic lateral sclerosis (ALS) and Parkinson’s disease (PD) (see [57], for a similar result based on acoustic coarticulatory measures obtained from speakers with multiple sclerosis (MS), PD, and neurologically healthy controls).

Speaking rates for groups representing each of the Mayo Clinic dysarthria types [33] were lower than the rates for neurologically healthy control speakers, but similar among the dysarthria groups [58] and diadochokinetic rates for speakers classified as having spastic and ataxic dysarthria were significantly slower than typical but not different from each other [59]. (Of note, in [59] a significant difference was obtained between the spastic and ataxic dysarthria groups in the variability of DDK sequences, with greater variability observed for the spastic group. This result, plus the statistically similar rates for the two disease/dysarthria groups, were contrary to the expectations from the Mayo classification system: Speakers with ataxic dysarthria were expected to have more variable speech rhythm than those with spastic dysarthria, and speakers with spastic dysarthria were expected to have slower rates than speakers with ataxic dysarthria. The authors comment that these findings require some reconsideration of dysarthria-type classification based on perceptual analysis). Vowel space areas for speakers with PD and ALS were statistically equivalent but smaller compared with neurotypical speakers [60]; second formant (F2) transition slopes were statistically equivalent in speakers with PD and stroke, but more shallow than slopes of neurologically healthy speakers [61]; voice-onset times for the three (English) voiceless stops produced in diadochokinetic sequences by speakers with the flaccid, spastic, ataxic, and hypokinetic types of dysarthria were similar across groups with the exception of /t/, for which speakers with ataxic dysarthria had longer VOTs compared to the other groups [48]. (These VOT data were derived from the well-known, original Mayo Clinic recordings from which the classification system was derived. There is no control group in the Morris study, and the variability of VOT measures was high, likely a result of variation in dysarthria severity and a combination of within- and across-speaker variability (each syllable within a 2.4 s sample from the full DDK sequence was measured for VOT, contributing multiple tokens for each speaker). In addition, the extent to which VOT values extracted from DDK sequences are representative of VOT measured in words in a carrier phrase, sentences, or read or spontaneous speech is another consideration for cautious interpretation of these results. See similar comments on the Morris [48] study in [62]).

Perceptual analyses of segmental errors in dysarthria are rare. One study compared vowel and consonant error patterns in Australian English of adults with the spastic and dyskinetic types of cerebral palsy and found no difference in the patterns except for greater severity among the dyskinetic group [63]. Speech intelligibility scores are not relevant to this discussion because they largely function as a metric of speech severity [64], which can vary in any disease/dysarthria type and therefore have little value in differentiating types.

Results of more recent publications are consistent with those of the aforementioned papers: speech production measures in speakers with dysarthria are typically different from those of control speakers, and infrequently different among disease/dysarthria groups. Studies consistent with this claim include (but may not be limited to) [7,47,50,52,53,55,65,66,67,68,69]. The inclusion of [47] in this claim is inferred from Figure 3 in [47], in which measures of F2 slope overlap considerably for eight different dysarthria “subtypes”. Correlation coefficients between maximum anterior tongue pressures and F2 slope were significant for the flaccid and mixed subtypes, but not significant for the other six subtypes. The results of [47] are discussed further in Section 6. Empirical Findings).

Substantial differences among disease/dysarthria groups in severity, speech material, presence or absence of a control group, and measurement type complicate direct comparisons among these studies. In some studies, variables such as speech intelligibility for different groups of speakers with dysarthria are not reported [47]. In at least three studies, relative speech severity, as measured by speech intelligibility scores or ordinal scale scores, was unbalanced across groups [50,65,70]. Across studies, speech materials from which measures were derived ranged from DDK [48] to single words [69], phrases [53], and passage reading [70].

Several of these studies included multiple measures (e.g., [71]), some of which were statistically different among groups whereas others were statistically equivalent. The present claim is that the weight of the evidence from the citations above favors a view that statistical similarities are more frequent than differences across multiple disease/dysarthria groups. Whether or not statistical significance was found for “better” (i.e., more sensitive) measures than those that were similar, or that one type of difference, such as of kinematic speeds, is more or less informative than, say, a measure that captures rhythmic characteristics across phrase level material, is unknown.

As suggested above, the speech production similarities among different disease/dysarthria types may reflect potential “core” features of speech production deficits in dysarthria [36,52]. The potential for dysarthria/disease-specific speech production characteristics is also likely, if not assured, given the ability of experienced clinicians and researchers to identify different dysarthria types even if not with a high degree of inter- and intra-listener agreement. In either case, it is not clear how performance on NSOM tasks can be designed to reveal similar and different phonetic deficits among disease/dysarthria types.

Three examples highlight the challenge of mapping oromotor nonverbal performance to speech production events. First, accuracy of vocal tract shape for vowels, rhotics, and laterals, as well as local perturbations to these shapes such as constriction location and geometry for obstruent production, are clearly important for segmental integrity and its effect on speech intelligibility (Refs. [52,72,73]; see discussion in [74], pp. 575–578). Moreover, how would an NSOM task be designed to shed light on whole-tongue curvature in vowel and rhotic production [75,76,77] or tongue shape and groove configuration for fricatives [78,79]? Second, what kind of oromotor nonverbal task (or quasi-speech task; see [26]), might capture the specific rhythmic structure of a language? Finally, how might stability of an oromotor nonverbal task map on to the many stabilities and variabilities in segmental and prosodic speech tasks? Variability in speech production occurs at many levels, including articulator positions for different consonants (e.g., Refs. [80,81,82]), coordination between two articulators for a particular segment and even across segments (coarticulation, also referred to as coproduction), sentence-level timing (see review in [83]), and the many acoustic phonetic outputs related to segmental and prosodic characteristics of the speech signal. Many of the articulatory variabilities for vowel segments such as /u/ and the lax vowels [84] and rhotics and liquids [85,86] are in the service of acoustic stability; in this sense, these are “good” variabilities. How might an oromotor nonverbal task be designed to distinguish “good” from “bad” variabilities, where “positive” variabilities are those in the service of segmental integrity and “negative” variabilities disrupt that integrity?

Suggestions have been made to design oromotor, nonspeech tasks relevant to speech motor control by creating tasks as similar to speech production as possible (e.g., Ref. [25]). In this view, oromotor non-speech capabilities are imagined to be arrayed along a continuum from gestures or efforts very different from speech motor control performance, to gestures and efforts very much like those observed in speech motor control, but without phonetic identity. Examples of the former include performance in maximum articulator strength, force stability, and rate of cyclic, non-verbal articulatory movement. Suggestions for the latter oromotor nonverbal tasks recognize the need to mirror the inherent time varying nature of phonetic gestures and their product, speech acoustic gestures [31,87], or the production of “silent” speech gestures (articulatory miming) such as raising the tongue tip to make contact with the alveolar ridge as in apical stops [25]. The relevance of a possible continuum of NSOM tasks to speech production behavior is discussed more fully in the next section.

## 4. Two Models of Speech Motor Control

### 4.1. Integrated Model (IM) versus Task Specific/Task Dependent Model (TSM/TDM)

Over the past six decades, many models or theories of speech motor control have been put forth (in the current paper, the terms “model” and “theory” are used interchangeably with full recognition of the important technical distinction between the two). Two models with implications for the relevance of oromotor nonverbal behavior to articulatory behavior are discussed here. The Integrated Model (IM) and the Task-Specific or Task Dependent Model (TSM, TDM) are at odds in this debate. They are worth discussing because they each have potential value in guiding the development of experimental hypotheses that are relevant to theories of speech motor control and clinical practice. Other, more formal (computational) models of speech motor control are available [28,88], and in fact, the IM and TDM share some assumptions with these models.

Models that differ in assumptions and content are tested against each other by contrasting predictions. The best-known example of prediction “contests” in speech science research pits the motor theory of speech perception against “auditory” theories of speech perception (for review, see [89,90]). In brief, the original formulation of the motor theory held that in humans, a species-specific, dedicated brain module served the function of speech perception [91,92]. The module does not use general auditory mechanisms in the brain to perceive speech. Rather, the module represents the objects-to-be-perceived as articulatory gestures—phonetic identities. These identities are automatic outputs of the module, and do not require transformations of the physical signal entering the central auditory pathways. The module, a product of evolution, is dedicated to the perception of speech and reflects the tight coupling in humans of speech production and speech perception. In contrast, auditory theories of speech perception suggest that general auditory mechanisms are employed to perform the perception of speech in humans, much as they are assumed to be for the perception of any auditory event; a special, species-specific analysis mechanism is not required.

The two theories of speech perception make opposing experimental predictions. The motor theory predicts that animals should not respond to variations in speech stimuli in the same way as humans—animals do not possess the special mechanism that would allow them to do so. In contrast, auditory theories predict the perception of speech signals is likely to be similar in both animals and humans because both use general auditory mechanisms to process the signals. This is a simplified statement of opposing predictions made by the two theories, but in general the predictions are consistent with their contrasting structure. Other theories of speech perception exist as well but are not discussed here; see [89,90].

This brief presentation of a well-known theoretical debate between special versus non-dedicated mechanisms in speech perception is directly relevant to the structure and predictions derived from the IM and TDM. The IM and TDM make opposing predictions as well, some as clear cut as the one just described for the case of speech perception. In this section, I show how the structure of the IM and TDM generate these predictions. To the best of my knowledge, previous publications on the nonspeech-speech issue have not described explicitly such predictions and how they derive from the respective structures of the models. Ironically it is possible the specificity of the opposing predictions presented below must be regarded as tentative because they are derived from two “models” that lack specificity.

The current structure of both the IM and TDM do not qualify as true models. More precisely, both are sets of statements that resemble assumptions, like primary premises in deductive reasoning. Both qualify as coarse-grained theoretical frameworks, with the proviso that a widely accepted definition of “theoretical framework” has been difficult to establish for research on speech motor control (see [93]).

When an SLP employs oromotor nonverbal or quasi-speech tasks to diagnose and/or treat a speech production deficit in a person with dysarthria, it is reasonable to ask, what are the reasons for this choice? Stated otherwise, what evidence is available and defensible for the effectiveness of oromotor nonverbal tasks in diagnosing a speech production disorder in dysarthria, or treating it with such tasks. (It is interesting that in the surveys that have been done on the use of oromotor nonverbal tasks in diagnosis and treatment of motor speech disorders, the data have focused primarily on whether or not the tasks are used, rather than why SLPs use them [15,22]. Models and theories, even if provisional, are important to how this question is answered. I do not discount the value of clinician experience in choosing a diagnostic or treatment approach. An SLP’s experiential database of therapeutic outcomes, and what seems to have caused (or not caused) them is important and should be included as a significant component of clinical decision making [94,95,96].

The structures of the IM and TDM are described first, followed by the predictions emerging from these structures. A later section describes how data in the literature related to these predictions support either (or both) theoretical frameworks.

#### 4.1.1. Structure of IM

The IM assumes, explicitly, that general motor processes are recruited for the production of speech. “Ziegler has interpreted our position as a *task-independent model* of motor control and in particular of speech motor control…we believe our model is better described as an *integrative model* in which some nonspeech motor tasks share principles with speech and some do not—that is, the speech motor system is integrated into a more general motor system. This leads us to postulate overlapping neural and behavioral systems for the control of speech and volitional nonspeech tasks” (Ref. [97] p. 38). Linguistic units such as phonetic segments are independent of motor processes; a domain-independent motor system transforms the units to action. By “domain-independent” motor processes, I mean motor control mechanisms that are not specialized for specific actions, such as (for example) speech production, playing an instrument, or finger spelling. Rather, the motor control capabilities of the nervous system are sufficiently powerful and flexible to be recruited and shaped for any and all actions. To be fair, Ballard et al. [97] allow that overlap of motor control for volitional, nonspeech and speech behavior is only partial, in that some nonspeech tasks are relevant to speech motor control, others are not. Which nonspeech tasks are not relevant to speech motor control are not suggested. Ballard, Granier, and Robin (Ref. [98] p. 983, emphasis added) put this view succinctly: “We rely on such theoretical stances as that presented by Folkins and Bliele (1990) and Saltzman (1986) which claim that the motor system is not necessarily organized around presumed units of language or speech. Rather, it is assumed that the motor system has its *own* cognitive architecture that is activated and monitored, in part, by the language system”. Speech motor control is therefore thought to be based on principles common to general movement control, regardless of what the movement is in service of. Similar views, though more nuanced for the case of speech motor control are presented in [99,100].

This nesting assumption, criticized for the case of speech motor control in [6], and more recent publications (e.g., Ref. [7]), drives the idea that speech motor control can be studied most directly by eliminating the speech acoustic signal that results from movement of the articulators, larynx and respiratory system. The speech acoustic signal is linguistic (e.g., Ref. [101]) and should be separated from evaluation of the movement control that generates linguistic events--phonetic behavior (e.g., voice onset time [VOT], vowel, diphthong, nasal, and semivowel formant frequencies), the various acoustic contributors to word and sentence stress, speech timing, and so forth. Stated otherwise, these “linguistic” events are not in the domain of speech motor control. In addition, the assumption eliminates, to a large extent, the relevance of movement type to understanding its control. Oromotor nonverbal tasks, whatever form they take, are stripped of the complexity of speech production movements (such as multi-articulator coordination over time and space) and therefore capable of allowing a basic analysis of effector control. In other words, such tasks are designed to move the analysis up to the top of the hierarchy—the general principles of motor control, free of specific goals.

It follows that IM rejects the idea that processes and perhaps the anatomy of speech motor control are modular (see [102,103]). It has been argued that “speech is not special”—echoing the theoretical difference between the motor theory of speech perception and auditory theories—and so does not require speech tasks to study and understand speech motor control (Ref. [26], p. 347). As argued below, the proper study of speech motor control requires tasks that include an acoustic phonetic, linguistically relevant product of underlying movements and forces, but in no way requires a specialized speech production neural module parallel to the one proposed in the motor theory of speech perception.

The IM also assumes that measures of speech motor control are well-served by measures of the parameters mentioned above (e.g., force, distance, tracking ability), in both oromotor nonspeech and speech tasks. Such measures have been used extensively in studies of finger, hand, arm, leg, and other non-oral motor behaviors, and in oromotor behavior as well.

Another assumption of IM is that a logical study of speech motor control, as well as other motor control tasks, is performed by isolating components of an ensemble of peripheral effectors that are organized to produce speech and other oromotor behaviors. This view is motivated by two explicit, supporting assumptions. One is that different orofacial subsystems—meaning the lips versus jaw, the jaw versus tongue, and lips versus tongue, the velopharyngeal port versus any of these structures “Subsystems” is used here following [8]. Rong et al. [104] have used the term to include the respiratory, phonatory, resonatory, and articulatory subsystems, which is more consistent with typical textbook descriptions of how the speech mechanism is partitioned [105]—can be differentially impaired in dysarthria. For example, speakers with dysarthria may have different degrees of impairment distributed across subsystems, often referred to as “differential impairment of the articulators” [103,106]. As discussed in Weismer [6], for this concept to make sense requires measurement of each isolated subsystem impairment, followed by a quantitative model that combines the results across the designated subsystems to estimate the likely deficit of the integrated, upper airway effector ensemble in the production of speech. The measurement of “isolated” subsystem impairment, seen through the lens of the IM, *must* be performed with oromotor nonspeech tasks, because in speech production, movements of the lips, tongue, and jaw do not function independently. Weismer (Ref. [6], p. 321) cited a passage from [107] endorsing this view. Subsystem movements of the lips, jaw, and tongue for speech production are coordinated over time and space, and, in the case of the jaw and tongue, and lower lip and jaw, are coupled anatomically and functionally, albeit in a loose-to-moderate way that depends on the speech sound (or syllable) production goal. It may be argued that tongue and lip movements can be isolated from movements of the jaw during speech, by fixing the position of the latter with a bite block. However, the matter of fixing the jaw in one position and therefore neutralizing it as a contributor to articulatory events is more complicated than it seems at first glance. Folkins and Zimmermann [108] showed that electromyographic (EMG) signals from jaw closer muscles were present for phonetic events requiring closure of the vocal tract, even when jaw position was fixed with a bite block. More recently, Dromey, Richins, and Low [109] reported the qualitative observation that, in their bite block experiment, some participants occasionally “bit down” on the block when they produced sentences. Although Dromey et al. did not specify when the participants did this, it would be interesting to know if the bitedowns were intermittent, possibly timed to phonetic events that in speech require closure of the vocal tract. Isolating the jaw, tongue, or lips during speech production, with all parts moving, does not seem to be possible, at least not currently. Weismer (Ref. [6], pp. 320–322; pp. 332–333) presents other significant problems with subsystems analysis. It should be pointed out that [104] performed an analysis of each component of more traditionally defined subsystems (respiratory, phonatory, resonatory, and articulatory) using multiple speech and speech-like measures. Rong et al. tested a tentative hypothesis for their participants with ALS that the measures for each subsystem represented its isolated impairment for speech production.

#### 4.1.2. Predictions from the IM

Predictions concerning speech production deficits follow from the structure and assumptions of the IM.

First, the IM predicts that a componential analysis of at least the three upper airway subsystems (tongue, lips, jaw) will provide useful information regarding a person’s oral communication skills. The prediction should apply to both speech intelligibility scores, as a “general” measure of speech deficit, and its underlying (“specific”, as discussed in Section 3.1) phonetic details, such as perceptually determined segmental errors [63] or automated estimates of segment “goodness” derived from the speech acoustic signal [110]. A challenge for this prediction is, as discussed above, the absence of a suitable metric of the overall deficit of orofacial muscular control based on componential analysis. This is so because the problem of “summing” the deficits of the three (or more) isolated parts and expressing the sum as an index of overall oromotor deficit has not been attempted and may not be conceptually feasible. (I am not arguing that the problem has zero potential to be resolved; “not attempted” does not strictly mean it has not been tried, but the absence of any such publications in the literature suggests either the absence of an attempt or, if attempted, a lack of success. However, a long-term research program can be envisioned to establish the proper way to express deviation from “normal” for each subsystem, perhaps using different tasks and establishing the best ones from the large number of potential oromotor, nonspeech gestures (see [6,25]). The research program could pursue formulae that combine subsystem component deficits that predict an overall, quantitative deficit of upper articulatory function for speech production, and even one that might account for specific speech sound deficits. How subsystem impairments that are quantitatively similar (e.g., 20% deviation from normal function for each subsystem) are combined for an estimate of overall oromotor deficit is a major issue that would need to be resolved. There is no a priori reason to expect 20% deviation from normal in a jaw oromotor nonverbal task to mean the same thing to speech production as a 20% deviation in tongue performance. A long-term goal would be to show that the quantitative nonspeech deficit maps onto speech intelligibility measures. I am not favorably inclined to this sort of work, because I think the effort would ultimately be futile. However, we all have our opinions, and in the end, a carefully developed proposal for this kind of work requires fair consideration; science is so much more valuable than opinion. Instrumental measures for the nonspeech behaviors would be far preferable for the work, but my use of “…perceptual and/or instrumental methods” is meant to acknowledge the often-used requests to patients to, “wag your tongue back and forth as fast as possible”, “open and close your mouth as fast as possible”, “press your tongue into your cheek as hard as you can, resist the pressure I apply”, and so forth. In Ziegler et al. [7], multi-task performance, including speech and non-speech tasks (by their definition DDK is considered a non-speech task), addresses a similar question by performing a statistical analysis on a large group of speakers with dysarthria and showing that speech and non-speech performance form mutually exclusive groupings).

Second, based on the continuum notion of NSOM tasks, ranging from very different from speech movements (e.g., steady force efforts exerted by each of the upper airway articulators, including the measurement of maximum forces or pressures, and/or endurance of maintaining maximum or submaximum forces), to movements that are similar to speech production gestures (e.g., soundless gestures like those observed for phonetic segments), performance on more speech-like NSOM tasks should predict speech intelligibility scores and phonetic “goodness” more accurately when compared with less speech-like tasks. A problem with this experiment is identifying how components (subsystems) of oromotor control can be partitioned for time-varying tasks, such as a soundless lingua-alveolar gesture.

The IM makes predictions for the treatment of neurogenic speech production disorders, which follow from the predictions outlined above for their diagnosis. For example, the componential assumption predicts that training of oromotor skills, in the absence of phonetic output of the vocal tract, will result in improved speech production skills either globally (e.g., improved speech intelligibility) and/or locally (e.g., at the segmental level of analysis). Implications of the IM for componential, oromotor training at the segmental level, such as training focused on the lips for improvement of labial consonant production or configuration for vowels, are (to the best of my knowledge) not considered explicitly in publications advocating the IM, but data have been published that are not consistent with the hypothesis [7,111,112].

Finally, a strong form of the IM predicts that therapy with a goal of improving articulatory skills in persons with dysarthria will be equally effective by training NSOM tasks versus NSOM tasks plus articulation, or NSOM tasks plus auditory training. The “NSOM task alone” condition would have to be paired with another form of non-oromotor training performance (e.g., a rhythm or reaction time task) to balance the conditions comparisons.

#### 4.1.3. Structure of the TDM

Ziegler [14] presents a “task-dependent model” (TDM) of speech motor control. The TDM contrasts with what he calls a “task-independent model”, which is similar if not identical to the IM. Stated otherwise, the “task-independent” model is consistent with the recruitment of general motor processes, as defined above, to implement the movements, forces, and sensorimotor mechanisms observable in speech production. In contrast, Ziegler’s task-dependent view is that speech motor control is implemented by motor processes unique to speech production. Ziegler argues that speech motor control is unlike motor control for non-verbal, oromotor actions such as those listed in a compendium published in [25].

The structure of the TDM, as currently developed, is coarse-grained in the same way as the IM. Like the IM, the model is best described by a series of statements, rather than by a more formal structure.

First, in the TDM, sensorimotor control processes for speech production are qualitatively different from those for oromotor control of non-speech actions. Motor control processes for the actions produced by structures of the speech mechanism are specific to speech production. In this view, the idea of a continuum of oromotor movements and configurations that vary in similarity to those required to produce speech does not make sense. Importantly, the sensorimotor processes in the TDM—that is, in speech motor control—include the speech acoustic signal and its integration with respiratory, laryngeal, velopharyngeal and articulatory movements, applied forces, and configurations. They also include sensorimotor processes for generating and regulating airflows and pressures and integrating them with movements and configurations. Sensorimotor processes in the TDM, including feedback from the speech acoustic signal and tactile and proprioceptive sensors (among other sensory information from structures of the upper airway, larynx, and respiratory system) are not separable from the gestures observed in speech production. They are an integral component of speech motor control [28,29].

Second, the TDM as currently conceptualized does not specify in *precise* neural terms how task dependency fits in with speech motor control processes. One possibility, discussed above, is that a dedicated nervous system network of nuclei and fiber tracts serves as the mechanism of speech motor control (i.e., it is a module). An alternative is that speech motor control may be vested in a learned and overpracticed “tuning” of interconnected nuclei and fiber tracts, developed from early childhood on, repeatedly refined by the millions of calibration samples and refinements that come with talking.

Third, the listener’s requirements for an intelligible speech signal enter the domain of, but not necessarily mechanisms for, speech motor control. These requirements are part of the goals of speech motor control, in the sense that they can dictate details of the task. Speaking metaphorically, the TDM allows, by alternate tunings of the neural substrate for oral communication, the speaker to know and adapt to what the listener needs [113].

Finally, the TDM is not consistent with the componential approach to the study of speech motor control. In the TDM, the goals of speech production—the production of acoustic signals useful for communication—are not accomplished by independent mappings of subsystem oromotor nonverbal performance to specific phonetic segments.

#### 4.1.4. Predictions of TDM

Several predictions emerge from the TDM that are opposite to those from the IM. First, the TDM predicts that measures of oromotor control for isolated structures such as the lips, tongue, and jaw, derived from non-speech actions,: will not predict an overall or detailed analysis of a speech production deficit, either summed across the structures or within single structures. A measure of verbal deficit in speech motor control is speech intelligibility, or other coarse-grained perceptual measures including but not limited to scaled estimates of severity. A “detailed analysis” might include perceptual evaluation of speech sound accuracy and prosodic characteristics, acoustic measures of different classes of speech sounds, measures positions, displacements, and velocities of movable upper airway structures or analysis of imaged tongue positions or configurations (such as ultrasound imaging: see Section 3.1). Stated otherwise, the TDM predicts that componential analysis will not sum to yield a variable that will predict speech intelligibility, or the accuracy of speech sounds, even when the evaluation of a specific, isolated component is matched to a speech sound group. For example, in the TDM perspective isolated evaluation of the tongue by means of oromotor, nonspeech gestures will not predict the correctness of tongue gestures for lingual consonants.

The TDM suggests several clinical predictions. For example, in a properly controlled experiment, therapeutic gains in speech production skills of persons with dysarthria should be greater among a group of individuals receiving speech production training, compared with persons trained to improve oromotor, nonverbal skills. An alternative version of this experiment is to compare therapeutic gains across two groups, one receiving oromotor, nonverbal training plus speech production training, the other speech production training plus training of a motor skill that does not involve oromotor structures, such as control of a measure of handgrip (e.g., target accuracy, or stability). The design of experiments such as these present challenges, many of which concern experimental control variables across groups such as comparability of severity, clinician competence, and stability of the underlying disease responsible for dysarthria. Assuming the experiment can be controlled, the concept of the latter experiment is probably closer to clinical reality than the former, which compares only oromotor, nonverbal training to speech production training.

Another prediction consistent with the TDM reverses the IM prediction that measures of oromotor, nonverbal control can be summed across subsystems to estimate the magnitude of a deficit in speech motor control. More precisely, estimates of overall speech motor control deficits, by speech intelligibility and/or severity scores, will not predict performance of oromotor, nonverbal control, summed across subsystems. A more fine-grained version of this prediction is that obstruent errors that differ in magnitude across place of articulation, will not predict the presence of differential weakness, displacement, speed, and so forth, in NSOM tasks that parallel the place-specific phonetic impairment.

### 4.2. Structure and Predictions from the IM and TDM: Summary

The IM and TDM have been discussed here because of their explicit application to theoretical and clinical understanding of motor speech disorders. Neither the IM nor TDM are true models; rather, both are a series of statements and assumptions that offer coarse-grained accounts of typical and impaired speech motor control in dysarthria (as well as in other speech deficits not covered here).

Selected experimental predictions derived by the current author from the structure and assumptions of the IM and TDM have been presented. Comparison of the predictions from the IM and TDM shows them to be in conflict. This is good for scientific and clinical communities, because the experimental evaluation of well-defined, opposing predictions should contribute to a qualitative, and eventually quantitative enhancement and refinement of either (or both of) the IM or TDM.

The IM and TDM have been described here as if separated by an impermeable conceptual wall. This is overly simplistic, but a starting point from which to determine experimentally if and how the two views can be melded (e.g., see [100]). Some formal models of speech motor control may represent execution goals in articulatory terms, such as vocal tract constriction location and degree, but the output of the model is an acoustic signal (Ref. [88], their Figure 1). Moreover, the speech acoustic signal is used to refine the shape, location, and degree of constriction—much as in Guenther’s [28] formal model. Nevertheless, a model with two (or more) simultaneous goals, even if not independent, may point the way to a hybrid model of speech motor disorders with IM and TDM principles.

## 5. Task Specificity

### 5.1. Task Specificity Introduction

An overarching issue in the potential link between oromotor nonverbal performance and speech production is the concept of task specificity, also referred to in the literature on motor speech disorders as task dependency. Task specificity, defined and discussed above as a critical difference in the motivating principles underlying the IM and TDM, is intertwined with the roles of common effectors, componential analysis of the mechanism of motor control, and functional goals in speech motor control.

### 5.2. Dystonia as a Task Specific Disorder

The concept of task specificity is not unique to speech motor control and its disorders. Focal dystonia, a motor control disorder in which unwanted, sustained muscle contraction results in distorted positions and postures of the trunk, legs, and arms, as well as cranial structures, may occur for a single effector and be restricted to one action (i.e., task). Examples of task-specific motor disorders include writer’s cramp and oromandibular dystonia. The single effectors in these disorders—the hand in writer’s cramp, the jaw in oromandibular dystonia—are manifest by involuntary, sustained contractions upon performance of a specific action. Writer’s cramp, in which the hand cramps immediately (or nearly so) when writing begins, does not affect the performance of other actions for which the hand is the primary effector [114]. Oromandibular dystonia is associated with involuntary, opening, closing, and sideway movements of the jaw, and in some cases, twisting movements of the lips and tongue. The involuntary muscle contractions are triggered by movements of the jaw and/or speech; the symptoms are often absent during sleep [115]. Both of these focal dystonias may be accompanied by tremors. Spasmodic dysphonia (SD) is regarded as a focal laryngeal dystonia, sharing with other focal and generalized dystonias the apparent absence of a motor disorder in certain tasks, and severe motor deficits in others. For example, in SD, the vocal folds appear on laryngoscopy to be normal and symmetrical at rest (during rest breathing), but during phonation for speech undergo or are subject to spasm-like contractions that interrupt the production of consonants and vowels. In other words, the clinical presentation of spasmodic dysphonia includes task specificity, that is, spasms triggered by speech production, but not other phonatory behaviors such as laughter and yawning [116]. Reference [32] included spasmodic dystonia as a dysarthria subtype of “hyperkinetic dysarthria”. Studies of the speech/voice production characteristics of hyperkinetic dysarthria are not frequent, possibly due to the relative rarity of dystonia compared with neurological diseases underlying other subtypes of dysarthria (e.g., Parkinson disease and hypokinetic dysarthria; upper motor neuron disease and spastic dysarthria). In a review of studies of isolated dystonia and dysarthria, published between 1976 and 2020, ref. [117] identified approximately 30 papers, 22 of which concerned persons with spasmodic dysphonia. Three papers reported data for persons with cervical dystonia; a single study of dysarthria in oromandibular dystonia was found. A reasonable conclusion is that task specificity is a familiar observation in neurological disease and in some cases is a requirement for certain diagnoses.

Focal dystonias highlight the importance of action goals, the latter integral to motor control. The goal is an integral part of motor control, not an independent, subtractable result of it. Stated otherwise, action goals are integral to motor control rather than an external result of it. Effectors are embedded in action goals; goals are not embedded in effectors.

### 5.3. Motor Learning and Task Specificity

Recently, the motor learning literature has seen a rapid growth of publications in which complex skills are studied. A recurring theme in this literature is the question of best training methods for skill acquisition and/or refinement. A frequent comparison is the training of basic motor capabilities, such as strength, or speed, to functional training in which the target skill is practiced. A recent review of the role of task specific, action control for hand positions, during tasks such as reaching, makes the case for a network of neural circuits within which the relative weights of neural activity in multiple networks are adjusted depending on the current hand position and its role in achieving a goal [118]. Successful accomplishment of the goal—such as grasping a cup—depends on movement control of the hand, wrist, and eyes. The authors] argue against decomposing the act of reaching into “parts”, such as eliminating visual input from an experiment to “isolate” the control of movement required for a reaching goal. Refs. [119,120,121] present similar views for task-specificity of motor control for actions other than speech at neural, physiological, and behavioral levels of analysis.

Rehabilitation of motor control disorders resulting from stroke and other acquired neurological disorders appears to be more efficient and effective when therapy is geared toward task-specific practice. As noted in [122], meaningless, repetitive actions, such as extensive practice of limb tone control, induce less effective improvement than practice of specific tasks that have meaning for an individual, such as walking. In animals, task specific training has been shown to induce more extensive cortical reorganization compared with repetitive tasks such as strengthening and tone control. Refs. [123,124,125] are additional sources for the superiority of task-specific versus non-task specific approaches in training motor skills in general as well as rehabilitation training for adults with acquired neurological disorders; see also [126].

### 5.4. We Are Talking about Task Specificity

Task specificity in neurologically typical and disordered speech motor control is a reasonable, if not obvious claim, not only based on the evidence reviewed above from the nonspeech, non-oromotor literature, but also based on theoretical arguments and published data. One of the centerpieces of the IM, in fact, is contradicted by the incompatibility of the evidence from this literature with that centerpiece. Specifically, the IM invokes “general” motor mechanisms as the basis of speech motor control, thus justifying pieces of the perspective, such as componential analysis and separation of action outcomes from the underlying processes of speech motor control. This implicates the control of general motor mechanisms in the study of *any* motor behavior—why speech motor control should be special compared to other actions such as reaching, walking, and sports skills is not at all clear. Thus, the incompatibility of the IM with the task-specific nature of speech motor control is inconsistent with data from other types of motor control, in which task-specificity is clear. According to the IM logic, this should not be the case. For best understanding in the IM view, analysis of motor control for reaching, walking, grasping, and producing many forms of music—for example, piano, guitar, zither—should be by separation of the actions into components with reduction of analysis of componential performance to measures such as maximum strength. The goals of these actions, which are musical outcomes, should be eliminated from such analyses. A “strong” form of the IM is defeated by its own assumptions.

Maas [26], following closely arguments made in [97], questions the ability to distinguish between tasks such as “speech” and “nonspeech”. More broadly, he is skeptical of the ability to define what a task is, and by implication the ability to contrast them as separate variables in an experiment. He bases this position on what I believe to be misinterpretation and misrepresentation of statements made in the relevant literature.

For example, Maas [26] and Ballard et al. [97] view the concept of “task specificity”—they use the term “task dependency”—in typical and disordered speech motor control as connected with the idea that *speech is special*. Ballard et al. [97] state, “Thus, it may not be the case that speech motor control is part of a task dependent system (i.e., speech is not special), but rather built from the family of motor processes that makes differences in behaviors related to tasks emerge” (p. 46). Admittedly, the meaning of this quote, relative to the current debate, is not clear. It could be taken as support for a tunable network (“a family of motor processes”) that reflects flexibility in function, and so allows a non-special speech production module to control variations in speech production behavior. Maas’ view is more nuanced but leans heavily on the notion that proponents of the TDM (or similar views) *must* invoke a module-like plant for the production of speech. However, the “speech is special” notion, in which speech motor control is vested in a special brain module has never been much of a player in speech production theories. A target article in which the evolution and current human underpinnings of speech motor control, including laryngeal and articulatory behavior, presents a case for the ontogenetic, *functional* reorganization of motor loops during the development of vocal tract control [127]. The target article, thirty commentaries on it, and the authors’ response make no mention of the kind of special, dedicated speech module like the one proposed for the motor theory of speech perception. More generally, such a module—dedicated and impermeable to other functions—has not, to my knowledge, been proposed for speech production. Thus, the equation of a task-specific (task dependent) perspective on speech motor control with the requirement for a special module is a straw man. Calling into question the definition of speech tasks, what they are and how they are nearly impossible to identify and distinguish from one another, distracts from the genuine issues. The implication that a “special mechanism” is required for any variation on oromotor behavior that results in varying forms of acoustic output from the vocal tract, is completely—and wrongly—dependent on the assumption that the TDM requires a special speech production module for every speech production task It is a reductio ad absurdum argument.

### 5.5. Accounting for Task Specificity without a Special Module

What are the neural alternatives to speech motor control vested in a special mechanism? A clue from scientists who study motor control of limb and hand actions and their underlying control for complex acts is experimental evidence for networks that can be tuned to accomplish different goals, and even to accomplish the same goal by subtle changes in the tuning (e.g., the single reaching goal, initiated from different positions [118]: “…after approximately 30 years of studies on the reference frame’s representation for hand position and movement in the cerebral cortex, experimental evidences and theoretical studies favor the existence of multiple, simultaneous representations of reach plans in different, dynamic (that is evolving during the task), hybrid, and task-dependent representations”: see also [121].

To argue for performance across a continuum of tasks, ranging from oromotor, nonverbal tasks to speech performance does not invalidate a task-dependency perspective on oromotor control. As in motor control of the limbs, hand, and eyes, and in postural control, motor speech production requires a network that includes attention to a goal, to the *meaning* of its task. The task in speech motor control is to enact phonetic sequences via the speech acoustic signal, which along with sensorimotor information originating in receptors in oromotor tissues, are integral to the tuning of networks serving speech motor control. By definition, oromotor nonverbal tasks are performed without access to the speech signal, as well as somatosensory information derived from air flows and pressures that are modulated by opening and closing valves (e.g., at the larynx, velopharyngeal port, and different locations along the vocal tract).

As described by Hickok [128], the dual-stream model of language processing includes an “auditory-motor” zone in the brain that coordinates the transition between speech perception and motor control for speech as well as simpler forms of vocalization such as humming. The humming example, according to Hickok, shows that activation of the zone is not “speech-specific”. However, it is specific to the generation of sound by the vocal tract (as in the case of humming). Activity in the zone is also “motor-effective selective”, responding more to covert humming when compared to keyboard reproduction of simple melodies (Ref. [128], p. 61). The auditory-motor zone is not separate from speech motor control processes, rather it is essential to the processing of receptive and expressive language. When an oromotor task does not require the integration of auditory and motor information, it is not speech. Within the speech motor control network, and in other motor control networks, subtle differences in activity weights can account for variations in language behaviors, including speech production. Within the auditory-motor zone, the balance of activity between auditory versus motor neurons can be tuned [128] to adjustments in speech behaviors. Such shades of tuning can be hypothesized for modifications of speaking rate, clarity, accent simulation, imitation, and other variations of speech production skills, including speech production acts that are not tied to meaning, such as diadochokinesis (DDK). The notion of task specificity in oromotor control, especially in the debate concerning the relevance of oromotor nonspeech behaviors to speech motor control, does not require, as Maas [26] argues, qualitatively different, special control mechanisms for every variation in how speech is produced.

Ultimately, the most powerful persuasion in this debate is vested in relevant data. The next section presents, in condensed form, results from studies in which relationships between performance on oromotor nonspeech tasks and measures of speech production were explored.

## 6. Empirical Findings

### 6.1. Selection, Organization, and Interpretation of Evidence

This section presents a summary of selected representative results, primarily published in the last twenty years, of relationships between performance on oromotor, nonspeech tasks and measures of speech production, speech acoustics, and of perceptual variables such as scaled severity of speech impairment and speech intelligibility. Some studies explored relationships between two or more oromotor nonverbal measures, or between oromotor nonverbal and speech production measures only in neurologically healthy individuals; these are listed in Table 1. Speakers in the studies listed in Table 2 are primarily people with dysarthria, but a few studies of speakers with apraxia of speech are summarized as well, including both children and adults. Several studies published prior to Weismer [6] are included in the current summary, but those reported in Tables II and III of Weismer (p. 325; 330) are not repeated here.

A comment is in order on the strategy for selecting articles summarized in Table 1 and Table 2. Searches of relevant databases (e.g., PubMed, Science Direct, Google Scholar) and citations in recent reviews of oromotor performance and its relationship with speech production guided selection of publications included here. “Selection” is perhaps not a precise term, as my aim was to include *all* relevant publications, primarily from the last two decades. The term “selection” is used to acknowledge the possibility of failing to identify relevant publications.The search strategy was not constrained by the outcome of a publication. No claim is made that these searches adhered to the rules of systematic reviews.

The findings of studies listed in Table 1 and Table 2 were evaluated with the qualitative approach used in [6]. The fourth columns in both tables are headed “Overall Findings”, which are judged for each publication to be “Positive”, “Mixed” or “Negative”. “Positive” denotes a study in which a significant relationship (or more than one) was found between oromotor, nonspeech performance and speech production and or speech intelligibility measures. “Mixed” indicates studies in which some relationships were significant, others nonsignificant. A judgment of “Negative” was made for studies in which no significant relationships were found, or, when multiple comparisons were made and the vast majority were nonsignificant. Because of the different conditions, participants, measures of both nonspeech and speech, and type of experiment across the group of studies listed in Table 1 and Table 2, many different groupings can be imagined for presentation of the reviewed studies. I chose to group the studies by their qualitative summary as explained immediately above. Within each subgroup (i.e., Positive, Mixed, and Negative) studies are listed alphabetically.

The judgments of overall findings in Table 1 and Table 2 are subjective. I have tried to offset confirmation bias by conservative assignment of overall findings, especially in the case of “mixed” judgments. The tables are meant as a guide; different opinions may be formed than the ones given here.

Also available are several systematic and narrative reviews of studies relevant to the oromotor nonverbal/speech production debate. These are summarized first, followed by studies including only neurologically healthy people (Table 1). This section is concluded with the data from studies in which the participants were either persons with neurologically based speech disorders, or persons representing both neurologically healthy and neurologically based speech disorders (Table 2).

### 6.2. Published Reviews

A small number of narrative, scoping, and systematic reviews are available concerning the role of oromotor nonspeech performance in understanding both typical and disordered speech motor control. Ref. [129] reviewed the imaging and behavioral data concerning temporal control for speech production and nonspeech (e.g., finger tapping), and concluded that speech timing is a composite of speech-specific and general “clock” mechanisms. This review is relevant to questions of how rhythm and rate in speech production might be modeled by oromotor nonverbal tasks (e.g., Ref. [35]). Refs. [16,18,20,130,131] reviewed the use of NSOM tasks in treating speech sound (phonological) disorders in children, and their role in speech development. All of these reviews note the lack, even absence of, evidence for the effectiveness of oromotor nonverbal tasks in speech development and speech sound disorders. Lass and Pannbacker (Ref. [130], p. 408) concluded their review by stating that, “OMEs should ‘be excluded from use as a mainstream treatment until there are further data”. The systematic review by [20] of the role of oromotor exercises in the treatment of speech disorders identified eight articles that met the strict criteria for inclusion; none provided compelling evidence for the efficacy or effectiveness of these techniques. McCauley et al. (Ref. [20], p. 353) take a conservative view of their analysis: “The conclusion that must be drawn from this review is the existing research literature provides insufficient evidence to support or refute the use of nonspeech OMEs”. Thus, Lass and Pannbacker, and McCauley et al., both identify the absence of positive evidence, but reach different conclusions about how to interpret the findings. Kent [25] (p. 774), based on a narrative review, judges the existing evidence for a role of oromotor nonverbal performance in the understanding of typical and disordered speech motor control as “equivocal”: “Wholesale rejection of these methods seems imprudent, given their inclusion in apparently useful and efficacious assessments and interventions”.

In the present opinion, within the larger context of empirical findings prior to publication of [20,25] and the theoretical issues discussed above, the conclusion reached by Lass and Pannbacker [130] does not seem imprudent. Rather, the conclusion reflects a more prudent outlook on the available data than expressed by [20,25]. Negative effects do not always point to the need for more research or continued use in a therapeutic setting. The question can be asked, if oromotor, nonverbal performance seems so naturally relevant to typical and disordered speech motor control, why has it been so difficult to demonstrate that relevance experimentally, and in the clinic?

**Table 1:** Summary of studies of the relationships between oromotor nonverbal performance and measures of speech production in healthy individuals.

Authors/Date	Population	Tasks	Overall Findings	Comments
Chang, Kenney, Loucks, Polleto, Ludlow (2009) [132]	Healthy adults	Neural substrates of speech (e.g., /saip-kuf/) and non-speech gestures (e.g., kiss snort) (fMRI methods)	Positive	Activity regions for nonlexical, phonotactically legal “word” pairs overlap with pairs of oromotor behaviors
Tremblay & Gracco (2010) [133]	Healthy, right-handed adults	Comparison of neural substrates for volitional vs. stimulus-driven response selection for words and oral motor gestures	Positive	Largely overlapping neural network for selection of words and oral gestures
Tremblay & Gracco (2009) [10]	Healthy, right-handed adults	Repetitive transcranial magnetic stimulation (rTMS) used to interfere with pre-SMA during selection of words and oral gestures	Positive	rTMS had similar impact on volitional planning of words and oral gestures
Lancheros, Jouen, Laganaro (2020) [134]	Healthy adults	Investigated dynamics of motor planning for nonspeech gestures matched to monosyllabic words and nonwords (EEG, ERP methods)	Mixed	Production of speech and nonspeech gestures recruited the same neural networks but the dynamics were distinct for speech and nonspeech
Stipancic, Kuo, Miller, Ventresca, Sternad, Kimberley, Green (2021) [135]	Healthy youngadults	Evaluated effects of periods of continuous chewing and speech on speech motor learning (8-syllable nonwords)	Mixed	Continuous chewing facilitated subsequent speech performance, but periods of continuous speech impeded subsequent speech performance
Arnold, MacPherson, Smith (2014) [136]	Healthy children (7–9 years) and young adults	Autonomic arousal associated with speech and nonspeech tasks	Negative	Speech tasks elicit greater autonomic arousal than nonspeech. Children and adults had similar autonomic arousal for speech tasks but children had higher arousal for nonspeech tasks than adults
Bilodeau-Mercure P. Tremblay (2016) [137]	Healthy young (18–39 years) and older (66–85 years) adults	Lip and tongue muscular strength, endurance, and sensitivity compared to speech rate and accuracy of syllable sequences	Negative	Only muscular endurance of lips associated with age-related changes in speech
Bonilha, Moser, Rorden, Baylis, Frideriksson (2006) [138]	Young healthy, right-handed adults	Functional magnetic resonance imaging (fMRI) during speech and non-speech movements	Negative	Speech recruited left inferior frontal gyrus, but not the insula; insular activation not observed when speech contrasted with non-speech
Poletto, Verdun, Strominger, Ludlow (2004) [139]	Healthy adults	Compared laryngeal vocal fold movement and muscle activity during speech (sylla-ble production) and nonspeech gestures (sniff, cough, throat clear)	Negative	Found different combinations of muscle activation for tasks related to respiration, airway protection, and speech
Simione, Fregni, Green (2018) [140]	Healthy adults	Applied transcranial direct current stimulation (tDCS) to jaw movements associated with speech, maximum syllable repetition, and chewing	Negative	tDCS effects are task dependent
Tremblay, Houle, Ostry (2008) [141]	Healthy adults	Examined transfer of learning on pairs of utterances matched on kinematic features	Negative	Speech learning failed to transfer to utterances with movements similar to those of trained utterances
Tremblay, Shiller, Ostry (2003) [142]	Healthy adults	Mechanical (somatosensory) perturbation of jaw during speech, ‘silent speech,’ and non-speech jaw movement	Negative	Adaptation of jaw movement to force perturbation for speech but not nonspeech

### 6.3. Studies of Oromotor, Nonverbal Measures and Speech Performance in Healthy Individuals

#### 6.3.1. Positive Findings

The twelve studies listed in Table 1 differ widely in experimental questions and methods. Three of the studies [10,132,133] were judged to have positive results. Using fMRI, the authors of a previous study [132] observed overlapping areas of activity for non-lexical, phonotactically legal consonant-vowel-consonant (CVC) pairs (English) and pairs of non-speech sounds such as “kiss-snort” and “cough-sigh”. The non-speech gestures activated a wider range of neural tissue compared to the CVCs, but the extent of common activation areas for the two types of gesture led the authors of [132] to argue for shared mechanisms of vocal tract control in speech and oromotor nonspeech production. The authors of [10,133], using repetitive transcranial magnetic stimulation (rTMS) and fMRI, compared the neural mechanisms of response selection for speech (monosyllabic words) and oromotor nonverbal tasks (e.g., growling, kiss). Tremblay and Gracco argued that the results of the two studies suggest similar oromotor control for speech and nonspeech vocal tract sounds. In a general sense, their results and interpretation are consistent with [132], but the former work [10,133] was concerned with the commonalities of response selection mechanisms for the selection of speech versus oromotor nonspeech production, the latter [132] presumably with the overlapping topology of activity areas in the brain for speech and nonspeech sounds.

#### 6.3.2. Mixed Findings

The results of [135] (Table 1) were judged to be mixed. There was a positive effect of chewing gum for ten minutes on a subsequent, challenging speech motor learning task. Conversely, a ten-minute period of continuous speech resulted in negative speech motor learning relative to a control condition. The cortical silent period, the interruption of an EMG signal following TMS stimulation of a target location on the primary motor cortex, was not significantly different for lip electrodes across the continuous chewing and speaking tasks. The worse-than-baseline effect of continuous speaking, plus the absence of an electrophysiological effect, were judged to be negative results for the effect of chewing on speech production behavior. The interpretation of this study is complicated by the potentially variable effects of memory on the outcome and the counterintuitive difference between the chewing and speech conditions. Results from [134], an EEG/ERP study of nonspeech gestures, nonsense words and words, were judged as mixed due to findings of similar planning processes, but differing dynamics (the temporal evolution of ERPs) between nonspeech and speech tasks. The authors suggest these results are not consistent with the TDM but admit that the differing dynamics of the tasks—especially between nonspeech and speech—suggest modifications of the common processes as oromotor control nears the execution stage. Lancheros et al. seem to view the IM as linked inextricably with the “special mechanism” issue discussed in Section 4.1. Their notion of explaining the differing dynamics of a common network is very much like the idea of adjustable networks—not special mechanisms—in the service of differing action goals [118,143]. As noted above, special, dedicated mechanisms for speech production are not required in the TDM.

#### 6.3.3. Negative Findings

The remaining seven studies in Table 1 reported comparisons in which the negative outcomes favor a TDM-type model. Ref. [137] reported the effect of different oromotor, nonverbal tasks of the lips and tongue on rate and segmental errors in rapid syllable repetition across neurologically healthy groups of younger and older speakers. Among several oromotor, nonspeech behaviors such as lip and tongue strength, endurance, and tactile sensitivity, only muscular endurance of the lips was associated with age-related changes in speech. Using electromyographic recordings of laryngeal muscles [139], the authors investigated task-specific patterns of muscle activity for vocal fold vibration, cough, sniff, and rapid shifts between vocal fold opening and closing. According to the authors, their findings, “…limit the ability to use the same biomechanical models of muscle activity to predict movement across tasks” (p. 865). Ref. [136] found autonomic arousal in adults to be greater for speech compared to nonspeech behaviors, likely a result of the cognitive-linguistic nature of speech production compared to oromotor, nonspeech behaviors. A study of jaw kinematics under the influence of tDCS showed different effects in a speech task (sentence production) versus an oromotor, nonverbal task (chewing), suggesting task specificity for speech motor control [140]. In another study of speech jaw movements, participants learned nonsense utterances that had been paired with new (not learned) utterances constructed with expected movements similar to those trained. Transfer of learning of the trained movements to these new utterances did not occur (“speech learning is extremely local and incompatible with the notion that speech motor function involves a generalized dynamics representation” (Ref. [141], p. 2426). A similar study of movement corrections measured during jaw movement in response to force perturbations in a protrusive direction analyzed effects during overt, silent speech, and nonspeech gestures across conditions; similar corrections of jaw movement were observed for the overt and silent speech conditions, which differed from the nonspeech condition (Ref. [142], Figure 2, p. 886). Both Tremblay et al. studies involved a single articulator, which restricts the scope of interpretation. Finally, an imaging study showed significantly different regions of brain activity for speech versus nonspeech behavior (Ref. [138] in contrast to the findings of [132]).

The judgments above of study results in which speech and nonspeech behavioral, imaging, and electrophysiological analyses are compared and found to be different across tasks can be considered along with empirical data for neurologically healthy participants summarized previously by Weismer (Ref. [6] Table II). As noted above, the negative judgments were made when there was a difference between the speech and nonspeech tasks. The weight of the evidence for neurologically healthy individuals favors task specificity of speech motor control.

**Table 2:** Summary of studies of the relationships between oromotor nonverbal performance and measures of speech production in neurologically based speech disorders.

Authors/Date	Population	Tasks	Overall Findings	Comments
Berggren, Hung, Dixon, Bounsanga, Crockett, Foye, Gu, Campbell, Butterfield, Johnson (2018) [144]	Children with congenital myotonic dystrophy and healthy controls	Maximum anterior tongue pressure and lip strength, ratings of dysarthria	Positive	Moderate correlation of strength measures with dysarthria ratings
Jones, Crisp, Asrani, Sloane, Kishnani Kishnani (2015) [145]	Late-onset Pompe Disease	Quantitative analysis of lingual strength and speech intelligibility; judgments of dysarthria severity	Positive	As dysarthria increased, lingual strength decreased
Puyjarinet, Bégel, Gény, Driss, Cuartero, Kotz, Pinto, Dalla Bella (2019) [146]	Adults with Parkinson’s Disease	Compared orofacial (DDK, pseudoword), manual, and gait tasks	Positive	Reported general rhythmic impairment across structures
Bose & van Lieshout (2012) [31]	Adults with aphasia and healthy controls	Lip movement for speech-like and non-speech tasks (kinematic/coordination indices)	Mixed	No difference between bilabial closure kinematics for non-speech and bilabial DDK sequences, except at fast rates
Clark, Duffy, Strand, Hanley, Solomon (2022) [42]	Flaccid, spastic, mixed spastic–flaccid, ataxic, or hypokinetic dysarthria types and healthy controls	Compared orofacial muscle strength (tongue, lips, cheeks) across types of dysarthria and dysarthria severity	Mixed	Maximum strength and severity of dysarthria poorly correlated; Explains no more than 20% of variance across all speakers with dysarthria
Searl, Knollhoff, Barohn (2017) [147]	People with amyotrophic lateral sclerosis (ALS) and healthy adults	Lingual-alveolar contact pressure during speech; speech intelligibility	Mixed	Some measures correlated with word intelligibility; no significant group difference in %Max; varying bulbar severity may explain significant results
Tamura, Tanaka, Watanabe, Sato (2022) [47]	Patients with dysarthria and healthy adults	Maximum tongue pressure and speech intelligibility, DDK, second formant slope	Mixed	Maximum tongue pressure correlated significantly with F2 slope in males, not in females; No significant correlations between the maximum pressure and intelligibility or DDK rate
Whiteside, Dyson, Cowell, Varley (2015) [148]	Acquired apraxia of speech (AOS) and oral apraxia (OA)	Compared speech and volitional nonspeech oral movements	Mixed	Moderate association between AOS and OA but also evidence of double dissociation
Chu, Barlow, Lee (2015) [149]	Adults with Parkinson’s Disease and healthy controls	Perioral stiffness, labial movement amplitude, electromyographic activity during syllable production	Negative	No significant correlation between upper- or lower-lip stiffness with labial kinematics except for UL at fast rate
Dietsch, Solomon, Sharkey, Duffy, Strand, Clark (2014) [43]	Flaccid, flaccid-spastic, ataxic, hypokinetic, or spastic dysarthria and healthy controls	Perceptual and instrumental measures of orofacial muscle tone and different types of dysarthria; 3 studies	Negative	Negative in people with dysarthria; No clear relationship between dysarthria type and orofacial muscle stiffness
Mackenzie, Muir, Allen, Jensen (2014) [19]	Adults with post-stroke dysarthria	Randomized feasibility trial comparing speech practice alone to speech plus NSOM exercises	Negative	No difference in behavioral intervention outcomes
Neel, Palmer, Sprouls, Morrison (2015) [150]	Adults with oculopharyngeal muscular dystrophy and healthy controls	Articulatory tasks, tongue strength, speech-like tasks, perceptual speech ratings	Negative	Nonspeech tasks did not predict speech measures
Potter, Nievergelt, VanDam (2019) [151]	Children and adolescents with typical development, speech sound disorders (SD), motor speech disorders (MSD)	Tongue strength (IOPI) compared to severity of speech sound disorder	Negative	Tongue strength not related to severity of speech sound deficits in SD or MSD
Schölderle, Staiger, Ziegler (2018) [27]	Adults with cerebral palsy and concomitant cognitive impairment	Feasibility of speech and non-speech tasks in diagnosis of dysarthria	Negative	Speech tasks more feasible (no ‘dysexecutive’ behavior) relative to non-speech tasks
Solomon, Makashay, Helou, Clark (2017) [152]	Adults with dysarthria and healthy matched controls	Orofacial strength measures compared to speech intelligibility, articulation rate, fast-syllable repetition	Negative	“Tenuous links between orofacial strength and speech production disorder”
Staiger, Schölderle, Brendel, Bötzel, Ziegler (2017) [21]	Patients with neurogenic movement disorders (Parkinson’s disease, stroke, cerebral palsy, primary dystonia, progressive supranuclear palsy; cerebellar ataxia) and healthy controls	Rate data were compared for speech (oral reading), speech-like (rapid syllable repetition), and nonspeech (rapid single articulator) movements Factor analytic approach used to compare types of tasks	Negative	Factor analysis revealed that rate performance on different types of tasks loaded onto separate latent variables
Staiger, Schölderle, Brendel, Ziegler (2017) [34]	Patients with neurogenic movement disorders (Parkinson’s disease, stroke, cerebral palsy, primary dystonia, progressive supranuclear palsy; cerebellar ataxia) and healthy controls	Rate data were compared for speech (oral reading), speech-like (rapid syllable repetition), and nonspeech (rapid single articulator) movements Multiple single-case methods used to test differences between three tasks for individual patients	Negative	Statistically significant dissociations between rates on speech tasks and those from speech-like or nonspeech tasks for a number of patients
Wood, Hughes, Hayes, Wolf (1992) [153]	Patients with Parkinson’s disease, stroke, multiple sclerosis and healthy controls	Comparison of quantitative measures of lip force and clinical judgment of the presence of a motor speech disorder	Negative	Dissociation between force measures and presence vs. absence of dysarthria
Ziegler, Schölderle, Brendel, Risch, Felber, Ott, Goldenberg, Vogel, Bötzel, Zettl et al. (2023) [7]	Persons with dysarthria (PWD; 6 etiologies) and healthy controls	Evaluation of how a battery of nonspeech parameters relate to speech characteristics in PWD; 23 different diagnostic measures assessed	Negative	Standard orofacial motor tasks failed to characterize speech characteristics of PWD; Found lack of overlap between speech and nonspeech domains

### 6.4. Studies of Oromotor, Nonverbal Measures and Speech Performance in Individuals with Motor Speech Disorders and Other Selected Speech Disorders

Table 2 lists 19 studies in which comparisons were made between oromotor, nonverbal and speech tasks, for speakers with motor speech disorders and other selected speech and/or language disorders. The vast majority of these papers included speakers with dysarthria; a few studies reported data on speakers with aphasia, acquired apraxia of speech, and children with speech sound disorders.

#### 6.4.1. Positive Findings

Three studies were judged to show positive results for a relationship between oromotor nonverbal behavior and perceptual measures of dysarthria in children with congenital myotonic dystrophy and late-onset Pompe disease [144,145]. Anterior tongue strength was measured in Berggren et al. [144] using the Iowa Oral Performance Instrument (IOPI), a widely used device used to measure maximum anterior tongue pressures, and a force meter to measure lip strength via resistance of the lips to horizontal stretch. Dysarthria—presumably meaning, “severity”—was measured on a 4-point, rank order scale with numbers attached to descriptors such as “100% intelligible” and “reduced intelligibility”. It is not clear who made the number assignments on the intelligibility scale, or the reliability of the perceptual judgments. Berggren et al. (Ref. [144], p. 415) report, “The presence of any dysarthria showed a moderate negative correlation with IOPI performance…”. The significant correlation was −0.538: lower maximum tongue pressures were associated with higher numbers on the dysarthria scale. [145] reported a relationship between lingual strength and dysarthria severity in adults with late-onset Pompe disease. The statistical basis of this relationship is not correlative, but rather an observation that participants with greater tongue strength tended to have less severe dysarthria. The third positive results are from a study of persons with Parkinson’s disease [146], in which similar rhythmic variability was found across participants for three motor behaviors—gait, tapping, and DDK (rapid repetitions of /pʌtʌkʌ/). These findings were interpreted by the authors as a reflection of a domain-general timing mechanism. This positive evaluation is based on the absence of a clear dissociation between rhythmic control in speech and other motor behaviors.

#### 6.4.2. Mixed Findings

Five studies in Table 2 were judged to have mixed results, among which are four [42,47,147,148] concerning relationships between speech production tasks and oromotor, nonverbal measures of strength, tone, and movement. Ref. [42] obtained maximum strength measures for the lips, tongue, and cheeks for speakers with one of five types of dysarthria as reported in Table 2. The maximum strength measures distinguished some dysarthria groups from others, but the statistical effects depended on oromotor task (e.g., tongue protrusion, cheek compression, lip compression) and dysarthria type. Orofacial maximum strength measures were weakly correlated with perceptual estimates of dysarthria severity, at best explaining 20% of the variance between the measures. The judgment of mixed results is based on some discrimination of dysarthria type by maximum strength, but poor prediction by the latter measures of dysarthria severity. In a study of intercorrelations among measures of maximum tongue pressure, DDK rate, F2 slope for Japanese diphthongs and glides, and speech intelligibility in a group of speakers with dysarthria, only F2 slope in male participants was correlated significantly (r = 0.397) with the maximum strength values [47]. When the data were broken down by dysarthria type, the significant maximum pressure-F2 slope correlations were detected for speakers with flaccid dysarthria (r = 0.786) and mixed dysarthria (0.640). Examination of Figure 3 in [47] suggests a disproportionate role of severity in these statistical relationships. Ref. [148] related speech accuracy scores to nonspeech oral movements in adult speakers diagnosed with acquired AOS and found a significant but weak correlation (0.395) between the variables (see their Figure 1). A study of speakers with ALS and control speakers [147] reported significant correlations between single word intelligibility scores and both the maximum anterior-tongue nonspeech pressures and contact pressures measured for lingual-alveolar consonants during speech production. These positive results are offset by the extensive overlap of speech task contact pressures, for some phonemes, between control speakers and speakers with ALS. In other words, between-group intelligibility differences seemed to be associated with similar contact pressures for some phonemes. Of great interest are the low-contact pressure values for lingual-alveolar consonants relative to maximum anterior tongue pressures for speakers in both groups [147]. This finding raises important concerns about the value of information provided by measurements of maximum tongue pressure, and similar maximum efforts in articulators such as the jaw and lips. Ref. [31] performed a study in which action goals of a string of nonspeech vocal tract gestures were matched to the goals of repeated syllable sequences (/pa/); the goal for both sequences was lip closure, and the two gesture sequences were produced at normal and fast rates by neurologically healthy controls and speakers with aphasia. Lip closure gestures were indexed by labial kinematic measures of amplitude, duration, peak velocity, and several derived measures of coordination. The statistical results showed no effect for Groups (controls vs. aphasic individuals) and a single main effect for LL duration for the difference between speech and nonspeech gestures. Significant Interactions between the main effects and rate were most prominent, with greater differences between DDK and sequential lip closure gestures at the fast rate. The judgment of mixed results is based on the similarity of lip closure kinematics—the absence of main effects for speech versus nonspeech gestures—but significant interactions between the gesture types and rate (see Table 2).

#### 6.4.3. Negative Findings

The eleven studies listed in Table 2 with a negative judgment include nine with straightforward summary statements. Speakers with PD showed non-significant correlations between a measure of lip stiffness and labial kinematic measures [149]. Small and/or nonsignificant correlations between orofacial maximum force measures and measures of speech production, or perceptual estimates of severity or speech intelligibility, were reported for dysarthric adults with oculopharyngeal muscular dystrophy [150] for children with motor speech disorders, as well as with motor speech disorders secondary to Galactosemia [151], for adults with various types of dysarthria [152], and for speakers with PD [153]. Using instrumental measures, ref. [43] failed to find a relationship between instrument-based and perceptual measures of orofacial muscle tone and severity of dysarthria in groups of persons with the same dysarthria types as in [42]. In a series of studies [7,21,34] performance by speakers with dysarthria on oromotor nonverbal tasks of the type frequently administered in the clinic were grouped statistically in a different category compared with speech measures. In a related study [27], verbal speakers with cerebral palsy were significantly less likely to complete nonspeech (including rapid syllable repetition) than speech tasks (see also [112]; the opposite pattern—participants being less likely to complete speech but not nonspeech tasks—was rare. Finally, a randomized trial of speech improvement in adults with post-stroke dysarthria compared treatment outcomes between two groups, one of which received speech practice, the other speech practice plus oromotor nonverbal exercises; treatment outcome measures for the two groups were equivalent [19].

## 7. A Note on DDK

Kent, Kim, and Chen [13] recently reviewed the substantial literature on DDK up to the time of their publication. Four notable and worthy features of this paper deserve mention. First, the tabled citations and data are an invaluable resource for future scholarship. Second, separate sections are devoted to different disorders, and most importantly for the current essay, there is a carefully considered section on motor speech disorders (Ref. [13], pp. 597–604). Third, a graph is presented in Table 1 (p. 579) showing the yearly number of publications since 1990 in which DDK was studied or reviewed. In 2010, the number of relevant publications per year began to increase dramatically, reaching a peak of 46 publications in both 2020 and 2021. An educated guess projects a continuation of the trend for increasing publication on DDK in a variety of speech disorders, and especially in motor speech disorders. Fourth, most, if not all of the information in the section on motor speech disorders (and other sections) is concerned with DDK as a means to diagnose disease and/or dysarthria type, or childhood apraxia of speech or acquired apraxia of speech in adults, rather than identify specifics of speech production and their potential effects on speech intelligibility.

To the best of my knowledge, only a few studies have addressed the question of how DDK analyses inform knowledge of variables such as vowel and consonant precision, types of segmental or syllabic errors, even rhythmic characteristics of speech in the service of communication. Interestingly, two studies cited by [13], and earlier by [6], were concerned with the relationship between performance in a variety of NSOM tasks and segmental production in DDK strings. In his study of oromotor nonverbal performance by speakers with cerebral palsy and dysarthria, Schliesser [111] examined NSOM tasks that in theory might be linked statistically with segmental properties in the DDK strings, such as consonant place of articulation. This result was not confirmed by the expectation: “only one of the 15 intercorrelations possible between speech and nonspeech alternate motion rates seems noteworthy, repeating /gʌ/ with retraction and rounding of the lips” (Ref. [111], p. 262). A similar disconnect between oromotor performance and segmental production was noted in [112], and the authors observed that maximum rate of nonverbal lip contraction was not more highly correlated with the maximum rate of /pʌ/ repetitions compared with /tʌ/ repetitions, nor was the maximum rate of tongue contraction more highly correlated with the maximum rate of /tʌ/ repetitions compared with /pʌ/ repetitions. Analyses extended to possible implications of DDK characteristics (rate, variation of rate, precision of consonants and vowels) for segmental and/or suprasegmental characteristic of speech production would be helpful.

Measures derived from DDK performance may be used to track articulatory changes over time in persons with progressive neurological disease and dysarthria [104], or as a kind of stress test for oromotor integrity [154]. However, it is also possible that other measures, such as speaking rate derived from sentence production, can do the same with empirically derived limits for clinically important changes [155]. In a recent study [156], five acoustic measures derived from a sequential motion rate task, each measure hypothesized to correspond to a specific articulatory characteristic (e.g., F2 slope reflecting speed of articulatory movement; across repetition variability of VOT reflecting stability of speech sounds across repetitions) showed that taken together the measures classified five neurological *disease* types with “good to excellent” accuracy. An interesting extension of this work would be to see how these acoustic features mapped on to the same measures in connected speech samples.

DDK is a staple in our field but remains mysterious with respect to prediction of speech characteristics important to questions of speech intelligibility specifically, and communication ability generally. Apart from an index of severity, there is not a lot of evidence that it is useful for more specific observations. The opinion offered by [21] regarding the utility of DDK in clinical settings where people with motor speech disorders are diagnosed and treated seems correct until proven otherwise. The original recordings that served as the basis for the Mayo Clinic classification of dysarthrias provide a cautionary tale about the relationship between DDK and spontaneous speech. Each participant in the database produced several different speech samples, including DDK for stop-consonant-vowel syllables, as well as a spontaneous speech sample in conversation with the examiner. In these recordings, there are many examples of poor DDK performance within individuals, not only for rate but for rhythm and consonant precision, which nevertheless is associated with intelligible or minimally unintelligible speech during the spontaneous samples).

## 8. Overall Summary

SLPs and SLTs make frequent use of NSOM tasks, as do scientists who conduct research on speech production and its disorders. Yet a coherent theoretical treatment of, and rationale for, a meaningful relationship between the two have not been formulated and remain under spirited debate. A significant piece of the debate is the acceptance or rejection of the concept of task specificity, in which motor control processes depend on the goal of the action under control. Analysis of the structure and opposed predictions of two much-discussed “models” of oromotor control in nonspeech and speech motor control, the IM and TDM, were presented. The IM is critical of task specificity as a guiding principle in the study of speech motor control; the TDM embraces it. Information from the literature on limb and hand motor control, and on rehabilitation strategies to improve control in persons affected by acquired brain damage, was brought in to show how task specificity is a well-accepted and preferred concept in which goals are not separable from other motor processes. A critical distinction was made between NSOM tasks as a means to diagnose the type of neurological disease, compared with the prediction of speech production deficits likely to result in varying degrees of intelligibility loss, or intelligible speech production that calls attention to itself when listeners hear something different from “typical” speech patterns. Experimental results addressing the relationship were tabulated and analyzed, leading to the conclusion that there is little evidence for insights to specific characteristics of speech motor control deficits provided by oromotor nonverbal motor control tasks. The latter may have some use in diagnosing the neurological diseases responsible for a speech motor control deficit. However, the value of such information in identifying the speech movement and acoustic underpinnings of dysarthria in its many expressions has yet to be demonstrated. To move the research and clinical disciplines of *speech* motor control forward, the study of speech production and its goal of providing listeners with time-varying, linguistically relevant acoustic signals, is necessary and (I believe) sufficient.
